# Prevalence of azithromycin resistance after the COVID-19 era in clinical bacterial isolates from a tertiary care hospital in Gurugram, India

**DOI:** 10.3389/fmicb.2025.1585526

**Published:** 2025-04-28

**Authors:** Parbati Debnath, Md Fahim Alam, Manisha Khandait, Fohad Mabood Husain, Nayla Munawar, Aftab Hossain Mondal

**Affiliations:** ^1^Department of Life Sciences, Faculty of Allied Health Sciences, Shree Guru Gobind Singh Tricentenary University, Gurugram, India; ^2^Faculty of Medicine & Health Sciences, Shree Guru Gobind Singh Tricentenary University, Gurugram, India; ^3^Department of Food Science and Nutrition, College of Food and Agriculture Sciences, King Saud University, Riyadh, Saudi Arabia; ^4^Department of Chemistry, College of Sciences, United Arab Emirates University, Al Ain, United Arab Emirates; ^5^Department of Microbiology, Maulana Azad College, Kolkata, India

**Keywords:** azithromycin resistance, multidrug resistance, COVID-19, clinical bacteria, mphA gene

## Abstract

The increasing prevalence of antibiotic resistance in pathogenic bacteria poses a great healthcare problem worldwide. Azithromycin (AZM) is a very effective macrolide antibiotic to treat many bacterial infections, but increasing azithromycin resistance in clinical bacteria decreases the effectiveness of this vital antibiotic, which is a major concern. The primary aim of the present study was to investigate the prevalence of azithromycin resistance and the occurrence of mphA gene in bacteria isolated from various clinical samples in Gurugram, India. For this, 138 pure bacterial isolates were obtained from the Department of Microbiology, Faculty of Medicine and Health Sciences, SGT Medical College, Hospital & Research Institute, Gurugram, India, from February to June 2024. All the isolates were identified by VITEK 2 system, and *E. coli* (22.5%) was found to be the most common pathogen in urine samples. Screening for azithromycin resistance by agar dilution and minimum inhibitory concentration (MIC) method found 30 azithromycin-resistant bacterial isolates. The present study found the prevalence of azithromycin resistance in pathogenic bacteria from clinical samples is 22%, indicating an increase in prevalence after the COVID-19 era, which is a major concern. Antibiotic profiling data revealed that 100% of the azithromycin-resistant isolates were multidrug-resistant, which is a serious issue. Furthermore, plasmid-mediated mphA gene was successfully amplified by the PCR method from 11 bacterial isolates, which may be responsible for azithromycin resistance. Our findings indicate the rapid emergence of azithromycin resistance in pathogenic bacteria, highlighting the urgency of stringent surveillance and control measures.

## 1 Introduction

Antimicrobial resistance (AMR) serves to be one of the most critical matter of the 21st century. According to the World Health Organization (WHO), antibiotic resistance is a serious public health issue that requires immediate attention. By 2050, it is predicted that AMR will cause 300 million fatalities worldwide with approximately $100 trillion in financial losses (Davies et al., 2024). According to the Lancet report on the global burden of bacterial antimicrobial resistance in 2019, estimated 4.95 million deaths were associated with resistant bacterial infections. Presently, limited number of antibiotics are available to treat the diseases caused by pathogenic bacteria. Among the antibiotics, azithromycin plays very important role to treat various bacterial infections, including respiratory tract infections, typhoid, sexually transmitted diseases, and certain gastrointestinal infections (Dominic et al., 2025; Carey et al., 2021; Sharma et al., 2019; Steingrimsson et al., 1990). Azithromycin is a synthetic macrolide antibiotic of the second generation that has a broad spectrum antibacterial activity by preventing the synthesis of proteins in bacteria (Imamura et al., 2005). It mainly interacts with the 23S rRNA by binding to the bacterial ribosome’s 50S subunit and blocking the peptidyl-tRNA’s translocation (Zheng et al., 2020). The effectiveness of AZM as an antibiotic was facilitated by several advantageous pharmacological characteristics, such as acid resistance, a quick time to peak concentrations, and an 800-fold accumulation in phagocytes at the infection site and prolonged half-life, which permits a substantial oral dosage to sustain bacteriostatic activity in the diseased tissue for 4 days (Imperi et al., 2014). This antibiotic also possesses anti-inflammatory, immunoregulatory, and antibacterial modulatory effects that enhance its ability to treat infections and patients suffering with various respiratory tract inflammatory disorders (Zimmermann et al., 2018). Furthermore, it can enter the extracellular vesicles of bacteria, which are a form of secretory defensive mechanism (Heidary et al., 2022).

The development of azithromycin resistance in pathogenic bacteria against this important antibiotic is a serious issue. Azithromycin resistance develops through the excessive expression of efflux pumps, which pump the drug out of the cells, and mutations in the rrl gene domain V, which decrease the interaction affinity of azithromycin and may increase resistance (Pham et al., 2021; Zhang and van der Veen, 2019; Schmalstieg et al., 2012). Furthermore, bacteria possess several macrolide resistance genes (MRGs) that provide resistance through various mechanisms, including target modifications generated by rRNA methylases or encoded by erm genes, which are facilitated by phosphorylases, such as those encoded by mph(A) and mph(B) genes, or esterases, such as those encoded by ere(A) and ere(B) genes (Palma et al., 2017). Moreover, reports have reported that transferable genes encoding macrolide-efflux pumps include msr(A), mef(A), and mef(B), also responsible for providing azithromycin resistance (Cohen et al., 2017). In addition to the mentioned mechanisms, continuous selective pressure on bacteria due to self-medication and overuse of antibiotics is an important precondition for the development of resistance (Kolář et al., 2001). Azithromycin gained attention during the COVID-19 pandemic to treat SARS-CoV-2 infection due to the unavailability of proper therapy. The University of California, San Francisco, hosted the individually randomized, telemedicine-based clinical study “Azithromycin for COVID-19 Treatment in Outpatients Nationwide” (Schwartz and Suskind, 2020). Previous studies reported that 88% of people in some lower-middle-income countries self-medicated, and 38 million excess doses of azithromycin were used during the COVID-19 pandemic in India (Sulis et al., 2021; Quincho-Lopez et al., 2021). A study was reported from Kenya that azithromycin resistance increased significantly from before to after COVID-19, from 6.3 to 40.4%, and the macrolide mph(A) gene was shown to be the most prevalent AMR gene (Odundo et al., 2024). Azithromycin accounted for 24% of total antibiotic consumption in 2021, making it the most commonly used antibiotic in the population (Massarine et al., 2023). As a result, COVID-19 has altered the profile of AMR, necessitating immediate action to reduce the threat and maintain our ability to combat infections in the next decades (Abdelaziz Abdelmoneim et al., 2024). Unfortunately, as antibiotic resistance has grown over time, these drugs are becoming less effective in treating various diseases brought on by pathogenic bacteria in varied contexts (Benmessaoud et al., 2016). So, there is a current need to study the prevalence of azithromycin resistance among clinically important bacteria.

This study aimed to determine the prevalence of azithromycin resistance in clinical bacterial isolates in a tertiary care hospital in Gurugram, India, specifically to investigate outcomes after the COVID-19 era. Furthermore, the susceptibility pattern of azithromycin resistant isolates toward various classes of antibiotics was investigated. Moreover, the occurrence of mphA gene among azithromycin-resistant isolates was investigated by molecular methods.

## 2 Materials and methods

### 2.1 Bacterial isolates

All bacterial isolates were collected from various clinical samples including blood, urine, pus, sputum, stool, ETA aspiration, and cerebrospinal fluid (CSF) from patients received in the Medical Microbiology Laboratory, SGT Medical College, Hospital & Research Institute, Gurugram, India, from February to June 2024, were included in the present study. Furthermore, those bacterial culture plates prepared from a single sample of the individual patient were obtained from the Microbiology Laboratory and processed for pure culture. We excluded data from the present study of duplicate isolates detected from the same patient’s sample. For this, a single colony was taken from the obtained plate and streaked on different media plates, such as Luria agar (LA), MacConkey, and Brain Heart Infusion agar (BHI), and incubated at 37°C overnight. After incubation, the colony morphology of the grown bacterial culture was studied to ensure a pure culture and assigned a separate identity name for further study (Supplementary Table S1). Finally, all the pure bacterial isolates were aseptically transferred to separate LB or BHI broth and incubated overnight to prepare glycerol stock. Ethics approval for this study was taken from the institutional ethical committee, Faculty of Allied Health Sciences, SGT University, Gurugram, India (Ref. No. FAHS/IEC/2023-24/69).

### 2.2 Identification of bacterial isolates

All the pure bacterial isolates were characterized as Gram-positive or Gram-negative by the standard Gram staining method. Furthermore, Gram-positive and Gram-negative bacterial isolates were separately identified by the standard VITEK 2 system at the Medical Microbiology Laboratory, SGT Medical College, Hospital & Research Institute. For this, pure bacterial colonies were suspended in 0.45% saline, and the density of the culture was adjusted to 0.5 McFarland. Each isolate was examined using the proper bioMérieux API strips, which included both Gram-positive and Gram-negative bacterial API. After being automatically filled by a vacuum device, the card was sealed, placed inside the VITEK 2 reader-incubator module (with an incubator temperature of 35.5°C), and every 15 min, its kinetic fluorescence was measured. The ID-GPC database interpreted the data, and automatic results were obtained in the end.

### 2.3 Screening for azithromycin resistance

All the bacterial isolates were preliminarily screened for azithromycin resistance by the agar dilution method. For this, Gram-positive and Gram-negative bacterial isolates were separately streaked on azithromycin-supplemented BHI (8 μg/mL) and LA (32 μg/mL) plates, respectively, as per CLSI guidelines (2020). All the plates were incubated at 37°C for 24 h; then, those isolates that showed growth on azithromycin-supplemented media plates were considered as azithromycin-resistant. Furthermore, all the preliminary resistant isolates were screened by broth micro-dilution assay to determine their MIC for azithromycin following CLSI 2020 guidelines. In brief, pure colony of each test isolate was inoculated into 10 mL LB broth and kept in a shaker incubator for incubation at 37°C overnight and then adjusted O.D. to 0.5 at 600 nm; the cells were further diluted using the same medium to a concentration of 10^6^ CFU/mL. Then, each well of a 96-well microtiter plate was initially poured with 50 μL of MHB media, except Row 1, where 80 μL of MHB was added. Next, 50 μL of MHB was added from Row 2 to Row 12 to make the final volume 100 μL. Then, 20 μL of antibiotic solution mixed in row A to create final concentration of 256 μg/mL and dilutions were made in the MHB media using 2-fold serial dilutions, to create concentration gradient 256, 128, 64, 32, 16, 8, 4, 2, 1, and 0.5 μg/mL. Finally, 50 μL of the bacterial culture was added to each well till Row 11 to make a final volume of 100 μL in the microtiter plate and resulting in a final inoculum density of 5 × 10^5^ CFU/mL in every single well. The plates were sealed and kept in a shaker incubator for overnight incubation at 37°C, 180 rpm. The lowest concentration of azithromycin which inhibits the growth of tested bacterial isolates was considered as MIC (μg/mL). The CLSI guidelines 2020 specify the criteria for azithromycin resistance, which are MIC ≥32 μg/mL for Gram-negative and MIC ≥8 μg/mL for Gram-positive bacteria.

### 2.4 Antibiotic susceptibility test of AZM-resistant bacterial isolates

Antibiotic susceptibility pattern of the azithromycin-resistant isolates was determined by Kirby-Bauer disk diffusion method against various antibiotics such as ampicillin (AMP), amoxyclav (AMC), cefoxitin (CX), cefotaxime (CTX), cefuroxime (CXM), tobramycin (TOB), imipenem (IMP), chloramphenicol (C), ciprofloxacin (CIP), amikacin (AK), tetracycline (TE), colistin (CL), gentamicin (GEN), and azithromycin (AZM), as per CLSI guidelines (2020). For this, single colonies of the azithromycin-resistant isolates were inoculated in 10 mL LB broth and incubated in a shaker incubator at 37°C for overnight. The O.D. of the cells of different isolates was adjusted to 0.5 at 600 nm. Then, a sterile cotton swab was dipped into adjusted cell suspension of each isolate and was spread evenly on the MHA plate. After spreading, appropriate antimicrobial-impregnated discs (HiMedia, India) were placed on the surface of the inoculum containing MHA plates at a proper distance using sterile forceps. After incubation of 16–18 h at 37°C, the zone of inhibition (mm) was observed and measured for each antibiotic toward different isolates with the help of a scale. After analyzing all the disc diffusion data, the tested isolates were categorized as sensitive (S), intermediate (I), or resistant (R), according to the CLSI guidelines.

### 2.5 Detection of mphA gene in azithromycin-resistant isolates

Genomic DNA was isolated from all the phenotypically AZM-resistant isolates by boiling and phenol chloroform isoamyl (PCI) method. Furthermore, plasmid DNA from all AZM-resistant isolates was extracted by commercially available QIAprep Spin Miniprep Kit as per the manufacturer’s instructions. Isolated genomic and plasmid DNA used as a template for PCR amplification of mphA gene with specific primers. The gene specific forward primer (5′-GTGAGGAGGAGCTTCGCGAG-3′) and reverse primer (5′-TGCCGCAGGACTCGGAGGTC-3′) used for PCR amplification of mphA gene were obtained from a previously reported study (Phuc Nguyen et al., 2009). The PCR master mixture (100 μL) was prepared as follows: 78 μL of Milli-Q water, 10 μL of TE buffer, 2 μL MgCl_2_, 4 μL of dNTPs, 2 μL each of forward and reverse primer, and 2 μL of Taq polymerase. The PCR reactions were performed as follows: initial denaturation for 5 min at 95°C, denaturation for 1 min (95°C), annealing for 1 min (60°C), and extension for 1 min (72°C), final extension for 5 min (72°C) for 25 cycles. The final products of PCR were subjected to gel electrophoresis on a 1% agarose gel and later visualized by the Gel-doc instrument.

## 3 Results

### 3.1 Isolation and identification of bacterial isolates

A total of 138 bacterial isolates were isolated from urine (*n* = 39), blood (*n* = 37), sputum (*n* = 30), pus (*n* = 19), endotracheal aspiration (*n* = 8), stool (3), and CSF (*n* = 2) within 5 months in 2024 in SGT Medical College, Hospital & Research Institute, Gurugram, India (Table 1). Among 138 bacterial isolates, 107 were found to be Gram-negative and 31 were Gram-positive. Furthermore, VITEK 2 system analysis identified 90 isolates up to species level as *E. coli* (31), *Pseudomonas aeruginosa* (10), *Acinetobacter baumannii* (9), *Klebsiella pneumoniae* (8), *Staphylococcus epidermidis* (7), *Staphylococcus aureus* (7), *Citrobacter freundii* (5), *Klebsiella aerogenes* (4), *Proteus mirabilis* (3), *Salmonella typhi* (3), *Klebsiella oxytoca* (1), *Pseudomonas oryzihabitans* (1), and *Enterococcus faecalis* (1). Analysis of data unable to identified 48 isolates up to species level was characterized on the basis of biochemical properties and named as none pathogenic organism (NPO, 23), Gram-positive cocci (GPC, 15), none lactose fermenter (NLF, 4), late lactose fermenter (LLF, 3), and lactose fermenter (LF 3). The results of Gram staining and the VITEK 2 system are represented in Supplementary Table S1.

**Table 1:** Bacterial isolates obtained from various clinical samples.

S. no.	Clinical source of sampling	Male	Female	No. of bacterial isolates
1.	Blood	15	22	37
2.	Urine	10	29	39
3.	Pus	12	7	19
4.	Sputum	17	13	30
5.	Stool	2	1	3
6.	ET Aspiration	5	3	8
7.	CSF	1	1	2
	Total bacterial isolates	62	76	138

### 3.2 Screening for azithromycin resistance

**Figure 1:**
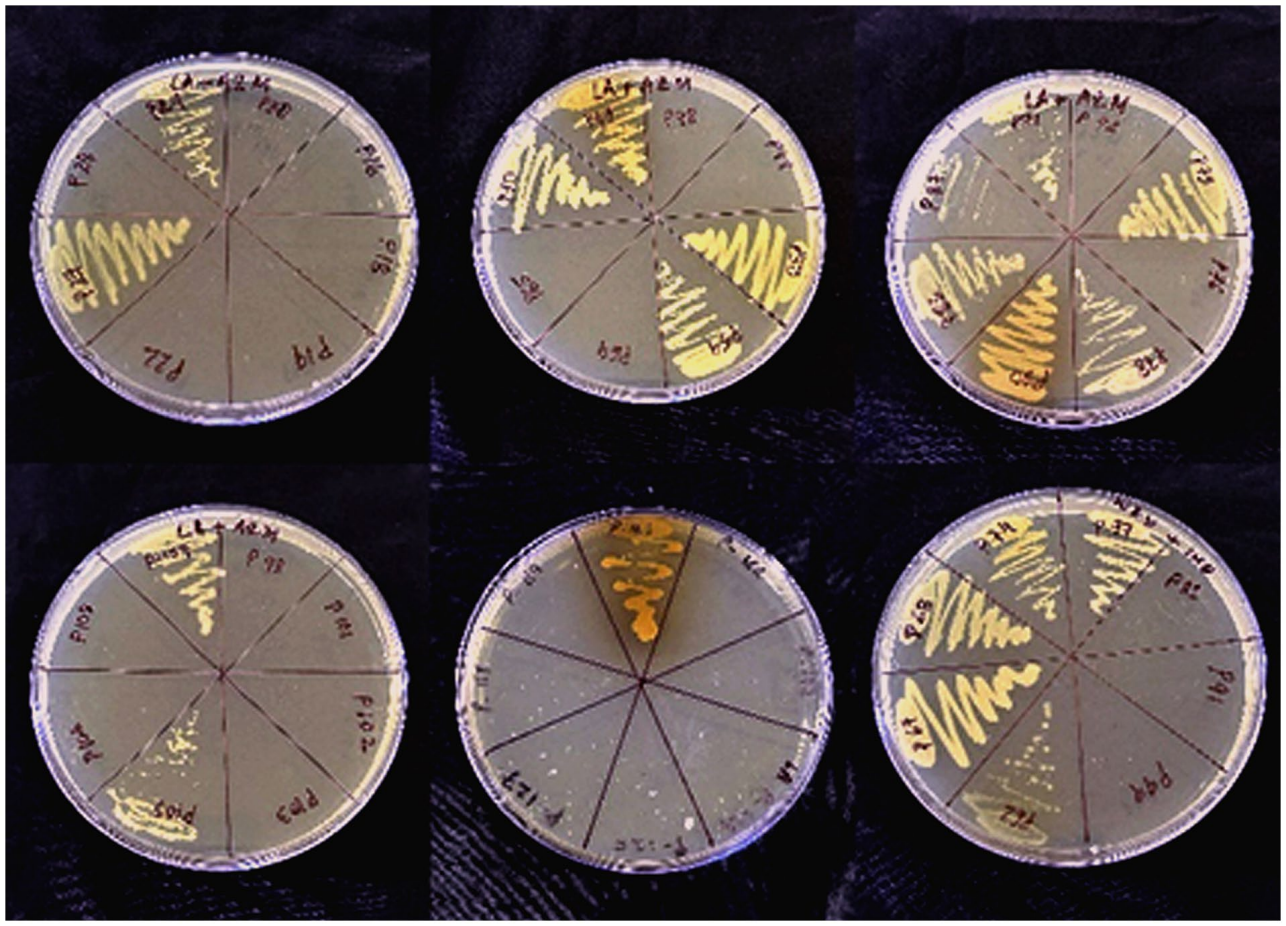
Visible growth of azithromycin-resistant bacterial isolates on azithromycin supplemented nutrient agar plates.

A total of 138 different bacterial isolates were screened for azithromycin resistance by agar dilution method. It was found that only 41 isolates grew on AZM supplementary media (Figure 1) and were preliminarily considered as AZM-resistant bacterial isolates. Among the tested isolates, high prevalence of azithromycin resistance was recorded for GPC (53%) followed by *K. pneumoniae* (50%), *Staphylococcus epidermidis* (42%), *Staphylococcus aureus* (42%), *E. coli* (29%), *A. baumannii* (33%), *P. mirabilis* (33%), and *S. typhi* (33%). Furthermore, analysis of all 41 isolates MIC data as per CLSI guidelines confirmed 30 isolates were resistant to azithromycin (Table 2). So, the overall prevalence of azithromycin-resistant bacteria from clinical samples is 22%. Among the isolates, high levels of MIC values were recorded against nine isolates >128 μg/mL, followed by six isolates 128 μg/mL, 14 isolates 64 μg/mL, and one isolate 32 μg/mL (Table 2). Among 30 AZM-resistant isolates, 24.19% (15/62) and 19.73% (15/76) were found from male and female patients, respectively (Table 3). Overall, high levels of AZM-resistant isolates were detected from CSF 50% (1/2), followed by ET aspiration 37.5% (3/8), Pus 31.57% (6/19), urine 25.6% (10/39), blood 21.62% (8/37), and sputum 6.6% (2/30) (Table 3).

**Table 2:** Minimum inhibitory concentration (MIC) of azithromycin against preliminary resistant bacterial isolates.

S. No.	Bacterial isolates	MIC (μg/mL)
1.	P4	4
2.	P6	4
3.	P55	64
4.	P120	64
5.	P20	128
6.	P68	128
7.	P80	64
8.	P71	64
9.	P73	64
10.	P29	64
11.	P109	64
12.	P27	64
13.	P32	64
14.	P128	64
15.	P5	128
16.	P9	8
17.	P14	8
18.	P19	16
19.	P42	64
20.	P70	128
21.	P78	4
22.	P137	128
23.	P47	64
24.	P11	>128
25.	P86	64
26.	P50	>128
27.	P121	>128
28.	P62	32
29.	P100	4
30.	P107	>128
31.	P74	64
32.	P134	>128
33.	P35	>128
34.	P34	>128
35.	P138	>128
36.	P52	>128
37.	P77	4
38.	P67	128
39.	P117	4
40.	P126	8
41.	P134	4

Green, susceptible; red, resistant.

**Table 3:** Source of all azithromycin-resistant bacterial isolates.

S. No.	Gender of patients	Samples	Bacterial isolates	Identified name
1.	M	Urine	P5	*E. coli*
2.	F	Urine	P11	*E. coli*
3.	M	Pus	P20	CONS
4.	F	Urine	P27	*P. mirabilis*
5.	F	Blood	P29	*A. baumannii*
6.	M	ETA	P32	*A. baumannii*
7.	M	Blood	P35	*S. aureus*
8.	M	Pus	P42	*E. coli*
9.	F	Urine	P47	*K. pneumoniae*
10.	M	ETA	P50	*K. aerogenes*
11.	F	ETA	P52	CONS P52
12.	M	Pus	P54	MG P54
13.	M	Pus	P55	*S. aureus* P55
14.	F	CSF	P62	GPC P62
15.	F	Blood	P67	GPC P67
16.	F	Blood	P68	GPC P68
17.	F	Sputum	P70	NPO P70
18.	F	Sputum	P71	LLF P71
19.	F	Urine	P73	*E. coli* P73
20.	F	Pus	P74	GPC P74
21.	M	Blood	P80	*A. baumannii* P80
22.	F	Urine	P86	*E. coli* P86
23.	M	Pus	P107	*S. aureus* P107
24.	M	Urine	P109	NLF P109
25.	F	Urine	P120	CONS P120
26.	M	Blood	P121	*C. freundii* P121
27.	M	Urine	P128	*E. coli* P128
28.	F	Blood	P134	GPC P134
29.	M	Urine	P137	*E. coli* P137
30.	M	Blood	P138	GPC P138

M, male and F, female.

### 3.3 Antibiotic susceptibility pattern of AZM-resistant bacterial isolates

**Figure 2:**
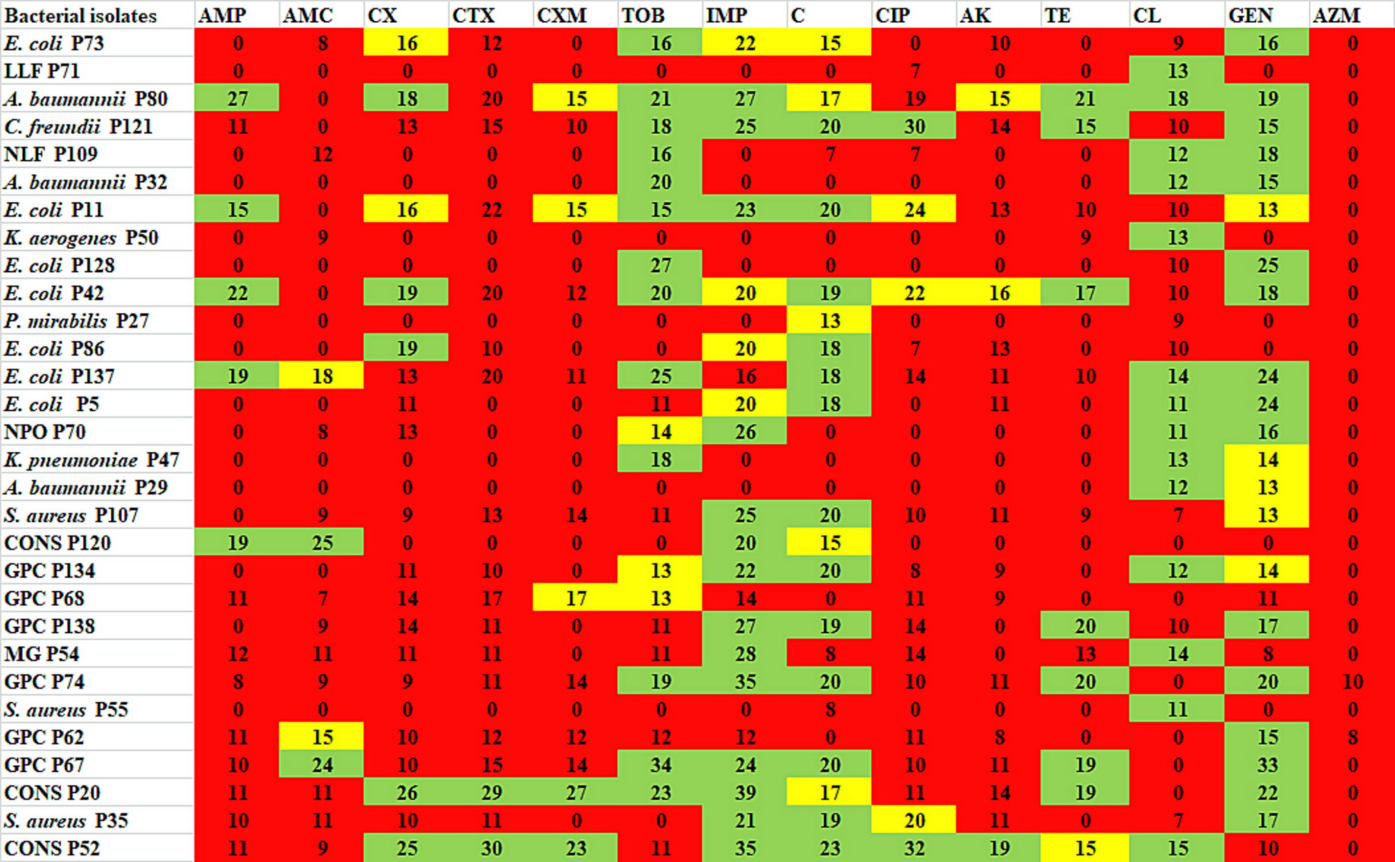
Antibiotic profiling of azithromycin-resistant bacterial isolates against different antibiotics presented as ZOI in mm scale. Green represents susceptible, yellow represents intermediate, and red represents resistant. AMP, ampicillin; AMC, amoxyclav; CX, cefoxitin; CTX, cefotaxime; CXM, cefuroxime, TOB, tobramycin; IMP, imipenem; C, chloramphenicol; CIP, ciprofloxacin; AK, amikacin; TE, tetracycline; CL, colistin; GEN, gentamicin; AZM, azithromycin.

**Figure 3:**
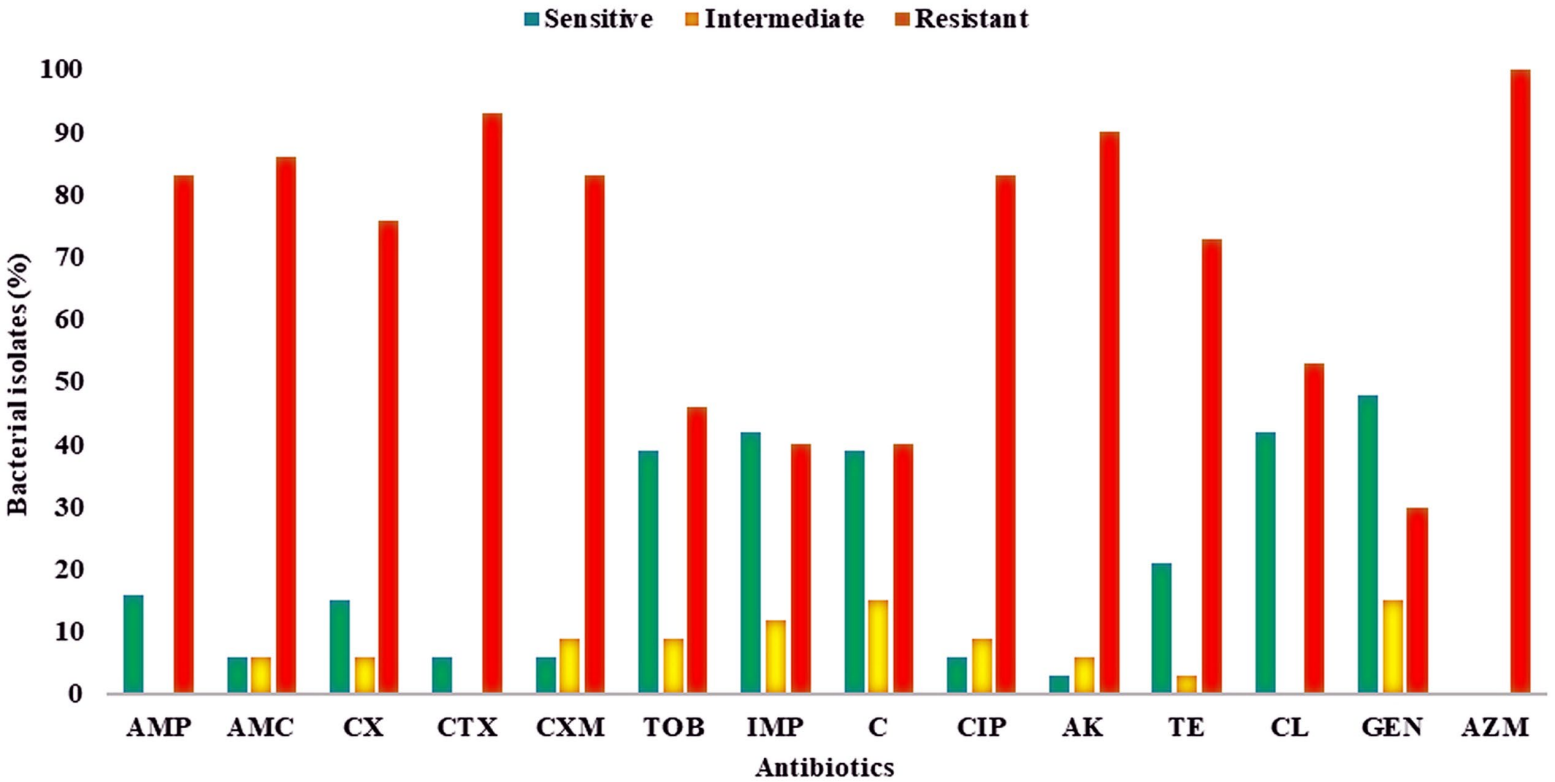
Antibiotic susceptibility of azithromycin-resistant bacterial isolates against different antibiotics, presented as percentages. Red, resistant; yellow, intermediate; green, sensitive. AMP, ampicillin; AMC, amoxyclav; CX, cefoxitin; CTX, cefotaxime; CXM, cefuroxime, TOB, tobramycin; IMP, imipenem; C, chloramphenicol; CIP, ciprofloxacin; AK, amikacin; TE, tetracycline; CL, colistin; GEN, gentamicin; AZM, azithromycin.

Antibiotic profiling of all 30 AZM-resistant bacterial isolates against 14 different antibiotics was investigated by the disk diffusion method, and the zone of inhibition was recorded in mm scale as shown in Figure 2. All the antibiotic profiling data were analyzed as per CLSI guidelines, and isolates were categorized as sensitive, intermediate, and resistant. All the tested isolates were found to be highly resistant against azithromycin (AZM) (100%) followed by cefotaxime (CTX, 93%) > amikacin (AK, 90%) > amoxyclav (AMC, 86%) > ampicillin (AMP, 83%) > cefuroxime (CXM, 83%) > ciprofloxacin (CIP, 83%) > cefoxitin (CX, 76%) > tetracycline (TE, 73%) > colistin (CL, 53%) > tobramycin (TOB, 46%) > imipenem (IMP, 40%) > chloramphenicol (C, 40%) > gentamicin (GEN, 30%), as shown in Figure 3. All the isolates were found to be highly resistant against most of the β-lactam and non-β-lactam classes of antibiotics except tobramycin, imipenem, chloramphenicol, and gentamycin, respectively. Multidrug-resistance (MDR) phenotype was observed among 100% of azithromycin-resistant bacterial isolates, which is a matter of concern. Furthermore, some isolates showed resistance toward more than 10 different tested antibiotics (Table 4). The antibiotic profiling data also suggested that imipenem (IMP), tobramycin (TOB), chloramphenicol (C), gentamicin (GEN), and colistin (CL) may be used to treat the infection caused by azithromycin-resistant bacteria.

**Table 4:** Antibiotic resistance pattern of azithromycin-resistant bacterial isolates.

S. no.	Bacterial isolates	Name of resistant antibiotic	No. of resistant antibiotic
1.	*E. coli* P5	AMP, AMC, CX, CTX, CXM, TOB, CIP, AK, TE, AZM	10
2.	*E. coli* P11	AMC, CTX, AK, TE, CL, AZM	6
3.	CONS P20	AMP, AMC, CIP, AK, CL, AZM	6
4.	*P. mirabilis* P27	AMP, AMC, CX, CTX, CXM, TOB, IMP, CIP, AK, TE, CL, GEN, AZM	13
5.	*A. baumannii* P29	AMP, AMC, CX, CTX, CXM, TOB, IMP, C, CIP, AK, TE, AZM	12
6.	*A. baumannii* P32	AMP, AMC, CX, CTX, CXM, IMP, C, CIP, AK, TE, AZM	11
7.	*S. aureus* P35	AMP, AMC, CX, CTX, CXM, TOB, AK, TE, CL, AZM	10
8.	*E. coli* P42	AMC, CTX, CXM, CL, AZM	5
9.	*K. pneumoniae* P47	AMP, AMC, CX, CTX, CXM, IMP, C, CIP, AK, TE, AZM	11
10.	*K. aerogenes* P50	AMP, AMC, CX, CTX, CXM, TOB, IMP, C, CIP, AK, TE, GEN, AZM	13
11.	CONS P52	AMP, AMC, TOB, GEN, AZM	5
12.	MG P54	AMP, AMC, CX, CTX, CXM, TOB, C, CIP, AK, TE, GEN, AZM	12
13.	*S. aureus* P55	AMP, AMC, CX, CTX, CXM, TOB, IMP, C, CIP, AK, TE, GEN, AZM	13
14.	GPC P62	AMP, CX, CTX, CXM, TOB, IMP, C, CIP, AK, TE, CL, AZM	12
15.	GPC P67	AMP, CX, CTX, CXM, CIP, AK, CL, AZM	8
16.	GPC P68	AMP, AMC, CX, CTX, IMP, C, CIP, AK, TE, CL, GEN, AZM	12
17.	NPO P70	AMP, AMC, CX, CTX, CXM, C, CIP, AK, TE, AZM	10
18.	LLF P71	AMP, AMC, CX, CTX, CXM, TOB, IMP, C, CIP, AK, TE, GEN, AZM	13
19.	*E. coli* P73	AMP, AMC, CTX, CXM, CIP, AK, TE, CL, AZM	9
20.	GPC P74	AMP, AMC, CX, CTX, CXM, CIP, AK, CL, AZM	9
21.	*A. baumannii* P80	AMC, CTX, CIP, AZM	4
22.	*E. coli* P86	AMP, AMC, CTX, CXM, TOB, CIP, AK, TE, CL, GEN, AZM	11
23.	*S. aureus* P107	AMP, AMC, CX, CTX, CXM, TOB, CIP, AK, TE, CL, AZM	11
24.	NLF P109	AMP, AMC, CX, CTX, CXM, IMP, C, CIP, AK, TE, AZM	11
25.	CONS P120	CX, CTX, CXM, TOB, CIP, AK, TE, CL, GEN, AZM	10
26.	*C. freundii* P121	AMP, AMC, CX, CTX, CXM, AK, CL, AZM	8
27.	*E. coli* P128	AMP, AMC, CX, CTX, CXM, IMP, C, CIP, AK, TE, CL, AZM	12
28.	GPC P134	AMP, AMC, CX, CTX, CXM, CIP, AK, TE, AZM	9
29.	*E. coli* P137	CX, CTX, CXM, IMP, CIP, AK, TE, AZM	8
30.	GPC P138	AMP, AMC, CX, CTX, CXM, TOB, CIP, AK, CL, AZM	10

AMP, ampicillin; AMC, amoxyclav; CX, cefoxitin; CTX, cefotaxime; CXM, cefuroxime, TOB, tobramycin; IMP, imipenem; C, chloramphenicol; CIP, ciprofloxacin; AK, amikacin; TE, tetracycline; CL, colistin; GEN, gentamicin; AZM, azithromycin.

### 3.4 Detection of mphA gene in azithromycin-resistant isolates

**Figure 4:**
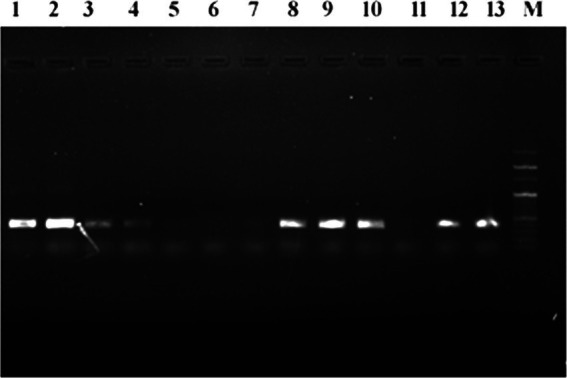
Agarose gel electrophoresis of PCR-amplified mphA gene products from plasmid DNA of various azithromycin-resistant isolates, M: 100 bp marker, 1–13 PCR amplified product of mphA gene.

Genomic DNA was successfully isolated from all AZM-resistant bacterial isolates and used as the template for PCR amplification of mphA gene, but no band for PCR products was observed in agarose gel after electrophoresis, indicating the absence of mphA gene in the genomic DNA of all AZM-resistant isolates. The plasmid DNA was successfully extracted from 17 different AZM-resistant isolates and used as the template for PCR amplification of the mphA gene. Figure 4 shows sharp bands of PCR amplicons confirming the occurrence of mphA gene in bacterial plasmid DNA. The mphA gene was successfully amplified from plasmid DNA of 11 different bacterial isolates including *E. coli* (6), *A. baumannii* (3), *K. pneumoniae* (1), and NLF (1) (Supplementary Table S2).

## 4 Discussion

The increasing prevalence of antibiotic resistance in pathogenic bacteria is a global concern, including India. Now, very limited numbers of antibiotics are available to treat the bacterial infections, but their effectiveness is sometimes compromised due to the development of resistance. Sensitive bacteria become resistant by various mechanisms; among them, continuous selective pressure due to self-medication as well as overuse of antibiotics is an important precondition for the development of resistance (Kolář et al., 2001). According to a systematic review, up to 88% of people in some lower-middle-income countries (LMICs) self-medicated with antibiotics to treat COVID-19 infection (Quincho-Lopez et al., 2021). Another study stated the sale of 38 million excess doses of azithromycin during the COVID-19 pandemic in India (Sulis et al., 2021). So, this high overuse of azithromycin may increase the prevalence of resistant bacteria. Therefore, the present study aims to determine the prevalence of azithromycin resistance in bacteria isolated from clinical samples in a tertiary care hospital in India.

In the present study, a total of 138 bacterial isolates were isolated from various clinical samples such as blood, urine, sputum, pus, stool, ET aspiration, and cerebrospinal fluid (CSF) in a tertiary care hospital in Gurugram, India. Among the isolates, 45% and 55% were obtained from male and female patients’ clinical samples, respectively. Generally, female patients are more prone to infection as compared to male patients due to anatomical and physical reasons. In the present study, the majority of bacterial isolates (77.5%) were found to be Gram-negative; generally, it is more prevalent compared to Gram-positive bacterial infections. Furthermore, VITEK 2 system analysis identified 65.21% isolates up to species level, and 34.78% isolates were characterized on the basis of their biochemical properties. Among the identified bacterial isolates, *E. coli* was found to be the most prevalent, followed by *Pseudomonas aeruginosa*, *Acinetobacter baumannii*, *Klebsiella pneumoniae*, *Staphylococcus epidermidis*, *Staphylococcus aureus*, *Citrobacter freundii*, *Klebsiella aerogenes*, *Proteus mirabilis*, *Salmonella typhi*, *Klebsiella oxytoca*, *Pseudomonas oryzihabitans*, and *Enterococcus faecalis*. A previous study reported that *E. coli* was the most common pathogen in clinical samples responsible for various infections in humans (Heidary et al., 2022). Studies have reported that Gram-negative bacteria such as *E. coli*, *Pseudomonas aeruginosa*, *Klebsiella*, *Acinetobacter*, and *Gram-positive Staphylococcus* spp. are commonly isolated from clinical samples (Adembri et al., 2020; Harbarth et al., 2007). Our VITEK 2 system results are in line with a previous study conducted by Lee et al. (2011), where they identified 60% of the clinical isolates up to species level by the VITEK 2 system, which was further confirmed by 16S rRNA gene sequencing. In another study, Ling et al. (2003) correctly identified 82% of the clinical bacterial strains at the species level by VITEK 2 system. The inability to identify the remaining isolates may be attributed to their non-fermentative nature or to potential errors in sample preparation during the VITEK 2 identification process.

In the present study, out of the 138 bacterial isolates examined by agar dilution method, 41 isolates were evaluated as azithromycin-resistant. Comparable results have been previously reported for both the E-test and the agar dilution approach (Papp et al., 2018; Gose et al., 2013), which supports the *E*-test’s use in surveillance programs to determine bacteria susceptibility to azithromycin. Specifically, in our study, we acknowledged that agar dilution was only done once for isolates, and even while we find this procedure repeatable for both clinical and quality control strains in our laboratory, the results could have been influenced by the quality of the medium. Furthermore, 30 isolates were determined as azithromycin-resistant by analysis of MIC values as recommended by CLSI 2020. So, the prevalence of azithromycin resistance in bacteria from clinical samples in Gurugram, India, is ~22%, indicating increased prevalence after COVID-19, which is the major concern. This finding is in line with a prior study that reported a significant increase of azithromycin resistance in *E. coli* and *Shigella* before to after COVID-19, from 6.3 to 40.4% in Kenya (Odundo et al., 2024). Studies have reported less prevalence of azithromycin resistance as compared to the present study in clinical bacterial isolates; those samples were collected before the COVID-19 era (Sharma et al., 2019; Parry et al., 2015; Challa et al., 2022; Lu et al., 2022). A prior study from India reported 93.2% of *S. typhi* and 76.7 % of *S. paratyphi* A were susceptible to azithromycin collected from the patients presented with enteric fever at the All India Institute of Medical Sciences (AIIMS) hospital, New Delhi, over a period of 25 years from 1993 to 2016 (Sharma et al., 2019). Not only in India, azithromycin has been reported as an effective antibiotic to treat *Salmonella enterica* serovar *typhi* and *paratyphi* A clinical isolates collected from seven Asian countries (Parry et al., 2015). Another study revealed that the percentage of *C. jejuni* resistant to azithromycin increased from 2% in 2019 to 4% in 2020 and 2021, while the percentage of *H. influenzae* resistant to azithromycin increased from 14% in 2019 to 52% in 2021 and dropped to 39% in 2022. None of the *S. typhi* isolates were resistant to azithromycin in 2019–2021, while 7% of the isolates were resistant in 2022 (Butt et al., 2024). A systematic review estimated the global prevalence of azithromycin in *Neisseria gonorrhoeae* on the basis of 134 reports from 51 countries over the past 30 years was 6% (Lu et al., 2022).

The high level of MIC values for azithromycin was recorded against nine isolates >128 μg/mL, followed by six isolates 128 μg/mL, 14 isolates 64 μg/mL, and one isolate 32 μg/mL. The most resistant bacteria *Klebsiella aerogenes* P50 had a highest MIC value of >128 μg/mL, *Proteus mirabilis* P27 (64 μg/mL), *Staphylococcus aureus* P55 (64 μg/mL), and late lactose fermenter (LLF) P71 (64 μg/mL), and also some of the isolates which showed least resistance with high MIC concentration were GPC P67 (128 μg/mL), CONS P52 (>128 μg/mL), GPC P138 (>128 μg/mL), *Staphylococcus aureus* P35 (>128 μg/mL), GPC P134 (>128 μg/mL), *Staphylococcus aureus* P107 (>128 μg/mL), *Citrobacter freundii* P121 (>128 μg/mL), *E. coli* P11 (>128 μg/mL), *E. coli* P137 (128 μg/mL), *E. coli* P5 (128 μg/mL), NPO P70 (128 μg/mL), CONS P20 (128 μg/mL), and GPC P68 (128 μg/mL). According to a North Indian study, between 2007 and 2016, the AZM MIC for *S. typhi* increased gradually from 8 μg/mL to 12 μg/mL (Munawer et al., 2020); in comparison with our study, there is a gradual increase in MIC for *S. typhi* which showed >128 μg/mL in 2024; this revealed a huge increase in MIC within 8 years. The current study evaluated a high resistance level in different antibiotics, for azithromycin (AZM) (100%) with extreme level of resistance, followed by cefotaxime (CTX) (93%) > amikacin (AK) (90%) > amoxyclav (AMC) (86%) > ampicillin (AMP) (83%) > cefuroxime (CXM) (83%) > ciprofloxacin (CIP) (83%) > cefoxitin (CX) (76%) > tetracycline (TE) (73%) > colistin (CL) (53%) > tobramycin (TOB) (46%) > imipenem (IMP) (40%) > chloramphenicol (C) (40%) > gentamicin (GEN) (30%). These antibiotic resistance patterns of azithromycin-resistant isolates are in accordance with previous studies in which the azithromycin-resistant bacteria showed high levels of resistance against different antibiotics (Katiyar et al., 2020; Jabeen et al., 2023; Chiou et al., 2023; Carey et al., 2021). Another study reported that Enterococci isolates have high levels of resistance (72–100%) to tetracycline, azithromycin, and ampicillin (Abera and Kibret, 2014).

In the present study, 93% of the isolates showed resistance against cefotaxime (CTX); a 3rd generation cephalosporin may be due to the ability of clinical bacterial isolates to produce beta-lactamase enzymes which break down the beta-lactam ring of CTX (Liang et al., 2016; Day et al., 2022). Those isolates that showed resistance against 3 or >3 different classes of antibiotics were considered multidrug-resistant (MDR). Surprisingly, all the azithromycin-resistant bacterial isolates have an MDR phenotype is the major concern. Extreme levels of resistance against 13 different antibiotics were found in *Proteus mirabilis* P27, *Klebsiella aerogenes* P50, *Staphylococcus aureus* P55, and late lactose fermenter (LLF) P71. From earlier investigations from Ethiopia, greater resistance levels were found in *S. aureus*, *Streptococcus* spp., *Proteus* spp., *Klebsiella* spp., and *Citrobacter* spp. when compared to the corresponding clinical isolates (Abera and Kibret, 2014). The present study evaluated that gentamicin (GEN) is the most effective antibiotic, followed by chloramphenicol (C) and imipenem (IMP) which can be kept aside. Another study from India conducted by Verma et al. (2024) assessed and found that the most effective medications for resistant infections are carbapenems, amikacin, and colistin. In India, one study investigated a rise in macrolide resistance, which discovered that every *Campylobacter* isolate is becoming resistant to macrolides and also exhibited a high level of resistance to azithromycin (Mukherjee et al., 2017).

The occurrence of mphA gene in bacteria, which encodes a macrolide 2′-phosphotransferase, has a significant role in inactivating azithromycin and inducing resistance. In the present study, genomic and plasmid DNA from azithromycin-resistant isolates were extracted to determine the presence of mphA gene by the PCR approach. We could not amplify mphA gene from the genomic DNA of azithromycin-resistant bacterial isolates, indicating absence of mphA gene in genomic DNA. Generally, the occurrence of mphA gene in plasmid DNA of azithromycin-resistant bacteria has been reported in a large number of previous studies (Asad et al., 2024; Wang et al., 2023; Xiang et al., 2020; Darton et al., 2018). In the present study, plasmid DNA was successfully isolated from 17 azithromycin-resistant bacterial isolates. The mphA gene was successfully amplified from plasmid DNA of 11 bacterial isolates, including *E. coli* (6), *A. baumannii* (3), *K. pneumoniae* (1), and NLF (1), which had high MIC values against azithromycin between 128 and >128 μg/mL. The mphA gene is always not present in all bacteria; therefore, rest of the bacteria may have different mechanisms for azithromycin resistance such as excessive expression of efflux pumps, and target modifications and mutations in the rrl gene domain V (Pham et al., 2021; Zhang and van der Veen, 2019; Schmalstieg et al., 2012). A previous study from India reported azithromycin minimum inhibitory concentrations (MICs) ranged from 4 to >256 mg/L in 28 of the 48 isolates that were investigated, which had the mphA gene (Mukherjee et al., 2017). According to the findings from earlier investigations, since *E. coli* is frequently carried on plasmids, the mphA can reside on both chromosome and plasmids, allowing it to spread broadly throughout the species that are closely related phylogenetically (Ma et al., 2017; Liang et al., 2017). According to the findings from Bangladesh tertiary care hospital, it was found that two *Salmonella typhi* isolates had azithromycin resistance genes for the first time: mphA (16.67%) and mefA (16.67%) (Dola et al., 2022). So, the presence of the mphA gene in different bacterial isolates could be the cause of the high incidence of azithromycin resistance. More studies are required to investigate the other molecular basis of azithromycin resistance in various pathogenic bacteria.

## 5 Conclusion

In the present study, the prevalence of azithromycin-resistant bacteria from various clinical samples is found to be 22% which is high as compared to previous study report before the COVID-19 era, which is the major concern (Sharma et al., 2019; Parry et al., 2015; Challa et al., 2022; Lu et al., 2022). This finding suggests that overuse of azithromycin during the COVID-19 pandemic might have created selective pressure for the development of resistance in the clinical bacterial pathogens. All the AZM-resistant isolates were also found to be resistant towards β-lactam and non-β-lactam class of antibiotics, and 100% of the isolates showed MDR phenotype, leading to a key problem. Furthermore, all the AZM-resistant isolates showed the highest susceptibility toward gentamicin (GEN), followed by chloramphenicol (C) and imipenem (IMP), and can be kept aside for future treatment. Molecular analysis revealed 64% (11/17) of isolates harboring plasmid-mediated mphA gene, which may be responsible for high levels of azithromycin resistance. More studies are required to understand the increasing prevalence of azithromycin resistance in pathogenic bacteria worldwide and the molecular mechanisms behind it.

## Data Availability

The original contributions presented in the study are included in the article/Supplementary material, further inquiries can be directed to the corresponding authors.
