# Supplementary material for: Prevalence of azithromycin resistance after the COVID-19 era in clinical bacterial isolates from a tertiary care hospital in Gurugram, India
Source: Front Microbiol. 2025 Apr 28;16:1585526. doi: 10.3389/fmicb.2025.1585526 (PMC12071464; doi:10.3389/fmicb.2025.1585526)
Supplement: Supplementary file 1 [file Table_1.docx]

Supplementary Material

# Supplementary Table

**Table S1:** Bacterial isolates from various clinical samples were identified by vitek-2 system.

| Sl. No. | Patients (M/F) | Clinical samples | Bacterial isolates | Gram staining | Name of bacteria |
| --- | --- | --- | --- | --- | --- |
| 1. | M | Blood | P1 | *Gram negative* | *Acinetobacter baumannii* |
| 2. | F | Blood | P2 | *Gram negative* | *Acinetobacter baumannii* |
| 3. | F | Blood | P3 | *Gram negative* | *Acinetobacter baumannii* |
| 4. | F | Blood | P4 | *Gram negative* | *Salmonella typhi* |
| 5. | M | Urine | P5 | *Gram negative* | *E. coli* |
| 6. | M | Pus | P6 | *Gram negative* | *E. coli* |
| 7. | F | Urine | P7 | *Gram negative* | *Pseudomonas aeruginosa* |
| 8. | F | Urine | P8 | *Gram negative* | *E. coli* |
| 9. | M | Sputum | P9 | *Gram negative* | *Pseudomonas aeruginosa* |
| 10. | M | Pus | P10 | *Gram negative* | *Pseudomonas aeruginosa* |
| 11. | F | Urine | P11 | *Gram negative* | *E. coli* |
| 12. | M | Urine | P12 | *Gram negative* | *Enterobacter aerogenes* |
| 13. | F | Urine | P13 | *Gram negative* | *E. coli* |
| 14. | F | Urine | P14 | *Gram negative* | *E. coli* |
| 15. | M | Sputum | P15 | *Gram negative* | *Klebsiella oxytoca* |
| 16. | M | ET Aspiration | P16 | *Gram negative* | *Acinetobacter baumannii* |
| 17. | F | Stool | P17 | *Gram negative* | *E. coli* |
| 18. | F | ET Aspiration | P18 | *Gram negative* | *E. coli* |
| 19. | F | Blood | P19 | *Gram negative* | *Klebsiella pneumoniae* |
| 20. | M | Pus | P20 | Gram positive | *S. epidermidis* |
| 21. | F | Pus | P21 | Gram positive | CONS |
| 22. | F | Urine | P22 | *Gram negative* | *E. coli* |
| 23. | F | Pus | P23 | *Gram negative* | *Pseudomonas aeruginosa* |
| 24. | M | Pus | P24 | *Gram negative* | *Pseudomonas aeruginosa* |
| 25. | M | Pus | P25 | *Gram negative* | *Pseudomonas aeruginosa* |
| 26. | F | Urine | P26 | *Gram negative* | *E. coli* |
| 27. | F | Urine | P27 | *Gram negative* | *Proteus mirabilis* |
| 28. | M | Blood | P28 | *Gram negative* | *Pseudomonas aeruginosa* |
| 29. | F | Blood | P29 | *Gram negative* | *Acinetobacter baumannii* |
| 30. | M | ET Aspiration | P30 | *Gram negative* | *Klebsiella pneumoniae* |
| 31. | F | Sputum | P31 | *Gram negative* | *Pseudomonas aeruginosa* |
| 32. | M | ET Aspiration | P32 | *Gram negative* | *Acinetobacter baumannii* |
| 33. | F | Blood | P33 | *Gram negative* | *Enterobacter aerogenes* |
| 34. | M | Blood | P34 | *Gram negative* | *Salmonella typhi* |
| 35. | M | Blood | P35 | Gram positive | *Staphylococcus aureus* |
| 36. | M | Sputum | P36 | *Gram negative* | *Klebsiella pneumoniae* |
| 37. | F | Pus | P37 | *Gram negative* | *E. coli* |
| 38. | M | Urine | P38 | *Gram negative* | *E. coli* |
| 39. | F | Pus | P39 | *Gram negative* | *Acinetobacter baumannii* |
| 40. | F | Urine | P40 | *Gram negative* | *E. coli* |
| 41. | F | Blood | P41 | Gram positive | CONS |
| 42. | M | Pus | P42 | *Gram negative* | *E. coli* |
| 43. | F | Urine | P43 | *Gram negative* | *E. coli* |
| 44. | F | Urine | P44 | *Gram negative* | *Klebsiella pneumoniae* |
| 45. | M | Blood | P45 | *Gram negative* | *Pseudomonas aeruginosa* |
| 46. | M | Blood | P46 | *Gram negative* | *E. coli* |
| 47. | F | Blood | P47 | *Gram negative* | *Klebsiella pneumoniae* |
| 48. | F | Blood | P48 | *Gram negative* | *Klebsiella pneumoniae* |
| 49. | F | Urine | P49 | *Gram negative* | *E. coli* |
| 50. | M | ET Transpiration | P50 | *Gram negative* | *Klebsiella aerogenes* |
| 51. | M | Blood | P51 | Gram positive | CONS |
| 52. | F | ET Aspiration | P52 | Gram positive | CONS |
| 53. | F | Urine | P53 | Gram positive | CONS |
| 54. | M | Pus | P54 | Gram positive | MG |
| 55. | M | Pus | P55 | Gram positive | *Staphylococcus aureus* |
| 56. | F | Blood | P56 | Gram positive | *Staphylococcus aureus* |
| 57. | F | Blood | P57 | Gram positive | GPC |
| 58. | F | Blood | P58 | Gram positive | GPC |
| 59. | F | Blood | P59 | *Gram negative* | *Salmonella typhi* |
| 60. | F | Urine | P60 | *Gram negative* | *E. coli* |
| 61. | M | Blood | P61 | Gram positive | GPC |
| 62. | F | CSF | P62 | Gram positive | GPC |
| 63. | M | Pus | P63 | *Gram negative* | *Proteus mirabilis* |
| 64. | M | Urine | P64 | *Gram negative* | *E. coli* |
| 65. | F | Urine | P65 | *Gram negative* | *E. coli* |
| 66. | F | Urine | P66 | *Gram negative* | *E. coli* |
| 67. | F | Blood | P67 | Gram positive | GPC |
| 68. | F | Blood | P68 | Gram positive | GPC |
| 69. | F | Sputum | P69 | *Gram negative* | NLF |
| 70. | F | Sputum | P70 | *Gram negative* | NPO |
| 71. | F | Sputum | P71 | *Gram negative* | LLF |
| 72. | M | ET Aspiration | P72 | *Gram negative* | *Pseudomonas oryzihabitans* |
| 73. | F | Urine | P73 | *Gram negative* | *E. coli* |
| 74. | F | Pus | P74 | Gram positive | GPC |
| 75. | M | Sputum | P75 | *Gram negative* | NPO |
| 76. | F | Sputum | P76 | *Gram negative* | *E. coli* |
| 77. | M | Blood | P77 | Gram positive | GPC |
| 78. | F | Sputum | P78 | *Gram negative* | NPO |
| 79. | M | Sputum | P79 | *Gram negative* | *Klebsiella pneumoniae* |
| 80. | M | Blood | P80 | *Gram negative* | *Acinetobacter baumannii* |
| 81. | M | Blood | P81 | Gram positive | GPC |
| 82. | M | Sputum | P82 | *Gram negative* | NPO |
| 83. | M | Sputum | P83 | *Gram negative* | NPO |
| 84. | F | Urine | P84 | *Gram negative* | *E. coli* |
| 85. | M | Urine | P85 | *Gram negative* | ASB |
| 86. | F | Urine | P86 | *Gram negative* | *E. coli* |
| 87. | F | Sputum | P87 | *Gram negative* | NPO |
| 88. | M | Sputum | P88 | *Gram negative* | NPO |
| 89. | M | Sputum | P89 | *Gram negative* | NPO |
| 90. | M | Stool | P90 | *Gram negative* | NPO |
| 91. | M | CSF | P91 | Gram positive | GPC |
| 92. | F | Urine | P92 | *Gram negative* | *Citrobacter freundii* |
| 93. | F | Urine | P93 | *Gram negative* | *E. coli* |
| 94. | M | Sputum | P94 | *Gram negative* | NPO |
| 95. | M | Sputum | P95 | *Gram negative* | NPO |
| 96. | M | Sputum | P96 | *Gram negative* | NPO |
| 97. | M | Stool | P97 | *Gram negative* | NPO |
| 98. | F | Sputum | P98 | *Gram negative* | NPO |
| 99. | F | Pus | P99 | Gram positive | *Staphylococcus aureus* |
| 100. | F | Blood | P100 | Gram positive | GPC |
| 101. | F | Urine | P101 | *Gram negative* | LF |
| 102. | F | Urine | P102 | *Gram negative* | *Pseudomonas aeruginosa* |
| 103. | M | Sputum | P103 | *Gram negative* | NPO |
| 104. | M | Blood | P104 | Gram positive | GPC |
| 105. | F | ET Aspiration | P105 | *Gram negative* | *Acinetobacter baumannii* |
| 106. | F | Sputum | P106 | *Gram negative* | NPO |
| 107. | M | Pus | P107 | Gram positive | *Staphylococcus aureus* |
| 108. | F | Sputum | P108 | *Gram negative* | NPO |
| 109. | M | Urine | P109 | *Gram negative* | NLF |
| 110. | M | Sputum | P110 | *Gram negative* | NPO |
| 111. | F | Blood | P111 | *Gram negative* | NPO |
| 112. | F | Sputum | P112 | *Gram negative* | NPO |
| 113. | F | Sputum | P113 | *Gram negative* | NPO |
| 114. | M | Sputum | P114 | *Gram negative* | NPO |
| 115. | F | Blood | P115 | *Gram negative* | NLF |
| 116. | M | Sputum | P116 | *Gram negative* | NPO |
| 117. | F | Pus | P117 | Gram positive | *Staphylococcus aureus* |
| 118. | M | Pus | P118 | *Gram negative* | *E. coli* |
| 119. | M | Urine | P119 | *Gram negative* | *Citrobacter freundii* |
| 120. | F | Urine | P120 | Gram positive | *S. epidermidis* |
| 121. | M | Blood | P121 | *Gram negative* | *Citrobacter freundii* |
| 122. | F | Blood | P122 | *Gram negative* | *Enterococcus faecalis* |
| 123. | F | Urine | P123 | *Gram negative* | *Citrobacter freundii* |
| 124. | F | Urine | P124 | *Gram negative* | *Enterobacter aerogenes* |
| 125. | F | Urine | P125 | *Gram negative* | *E. coli* |
| 126. | M | Blood | P126 | + | GPC |
| 127. | M | Pus | P127 | *Gram negative* | *Citrobacter freundii* |
| 128. | M | Urine | P128 | *Gram negative* | *E. coli* |
| 129. | F | Urine | P129 | *Gram negative* | LF |
| 130. | F | Blood | P130 | Gram positive | *Staphylococcus aureus* |
| 131. | F | Blood | P131 | Gram positive | GPC |
| 132. | F | Urine | P132 | *Gram negative* | *Proteus mirabilis* |
| 133. | M | Urine | P133 | *Gram negative* | *E. coli* |
| 134. | F | Blood | P134 | Gram positive | GPC |
| 135. | F | Sputum | P135 | *Gram negative* | *Klebsiella pneumoniae* |
| 136. | F | Urine | P136 | *Gram negative* | NLF |
| 137. | M | Urine | P137 | *Gram negative* | *E. coli* |
| 138. | M | Blood | P138 | Gram positive | GPC |

M, male and F, female.

**Table S2** PCR based detection of mphA gene in the plasmid DNA of azithromycin resistant bacterial isolates.

| - Sl. No. | Bacterial isolates | mphA gene |
| --- | --- | --- |
|  | *E. coli* P5 | **+** |
|  | *E. coli* P11 | **+** |
|  | *P. mirabilis* P27 | **-** |
|  | 1. *Baumannii* P29 | **+** |
|  | 1. *Baumannii* P32 | **+** |
|  | *E. coli* P42 | **+** |
|  | *K. pneumoniae* P47 | **+** |
|  | *K. aerogenes* P50 | **-** |
|  | *S. aureus* P55 | **-** |
|  | NPO P70 | **-** |
|  | LLF P71 | **-** |
|  | *E. coli* P73 | **+** |
|  | 1. *Baumannii* P80 | **+** |
|  | NLF P86 | **+** |
|  | *C. freundii* P121 | **-** |
|  | *E. coli* P128 | **+** |
|  | *E. coli* P137 | **+** |

+, present and - , absent
